# Study Regarding the Kinematic 3D Human-Body Model Intended for Simulation of Personalized Clothes for a Sitting Posture

**DOI:** 10.3390/ma14185124

**Published:** 2021-09-07

**Authors:** Andreja Rudolf, Zoran Stjepanovič, Andrej Cupar

**Affiliations:** 1 Institute of Engineering Materials and Design, Faculty of Mechanical Engineering, University of Maribor, Smetanova 17, 2000 Maribor, Slovenia; zoran.stjepanovic@um.si; 2 Mechanical Engineering Research Institute, Faculty of Mechanical Engineering, University of Maribor, Smetanova 17, 2000 Maribor, Slovenia

**Keywords:** kinematic 3D human-body model, sitting posture, Blender software, virtual prototyping, personalized clothing

## Abstract

This study deals with the development of a kinematic 3D human-body model with an improved armature in the pelvic region, intended for a sitting posture (SIT), using Blender software. It is based on the scanned female body in a standing posture (STA) and SIT. Real and virtual measures of females’ lower-body circumferences for both postures were examined. Virtual prototyping of trousers was performed to investigate their fit and comfort on the scanned and kinematic 3D body models and to make comparison with real trousers. With the switch from STA to SIT, real and virtual lower-body circumferences increase and are reflected in the fit and comfort of virtual and real trousers. In SIT, the increased circumferences are attributed to the redistribution of body muscles and adipose tissue around the joints, as well as changes in joints’ shapes in body flexion regions, which are not uniformly represented on the kinematic sitting 3D body model, despite improved armature in the pelvic region. The study shows that average increases in waist, hip, thigh, and knee circumferences should be included in the process of basic clothing-pattern designs for SIT as minimal ease allowances, as should, in the future, armature designs that consider muscle and adipose tissues, to achieve realistic volumes for kinematic 3D body models in SIT.

## 1. Introduction

In recent years, the use of computer-aided design (CAD) with various computational and analytical tools has played an important role in clothing design and customization. Three-dimensional design software has been mostly used to improve the garment design process through overcoming the limitations of ordinary 2D design methods by generating virtual 3D clothing prototypes. In addition, many researchers have attempted to develop 3D virtual garment prototypes using 3D body models and involving 3D human-body scanning in a static standing or dynamic posture, respectively, as the benefits of virtual prototyping have been recognized. All studies on scanning technologies for the standard scanning processes for effective data collection to study human body-size and -shape and defined protocols for automatic body measurements in static and dynamic anthropometry are focused on accurate 3D body models and the complexity of 3D human body-scan data modelling, with the purpose of measuring accurate body dimensions intended for sizing systems or the development of personalized clothes in a virtual environment [1,2,3,4,5,6,7,8]. The standard avatars in 3D CAD systems still have limitations, and cannot be exactly adapted to the many body figures, postures or body deformities required for personalized clothing prototyping, e.g., for the elderly, athletes, disabled people, pregnant women, workers, etc. Therefore, 3D scanning in the required body postures and generation of accurate 3D models of individuals is required [9,10,11,12,13,14,15,16,17,18,19]. Studies by Abtew M.A. et al. [20,21,22,23] focus on the development of comfortable and well-fitted bra patterns for customized female soft-body armour through the 3D-design process of adaptive bust on a virtual mannequin. These studies introduced a novel design technique for developing female adaptive bust volumes on a 3D female virtual mannequin. Their results showed that the proposed method reflected satisfactory fit and comfort, compared with 2D clothing-pattern designs. A co-design-based method for generating two-dimensional basic clothing-pattern designs, for physically disabled people with scoliosis and using three-dimensional virtual technology, was presented in work by Hong Y. et al. [24,25,26]. The design of functional garments for people with scoliosis or kyphosis, using computer simulation techniques, kinematic 3D body models and the CASP methodology, has been investigated in other studies [27,28]. CASP means curvature, acceleration, symmetry and proportionality. This methodology can be applied for the analysis of geometrical surfaces and their evaluation. An early stage of this analysis methodology was used in a study by Cupar et al. [29]. A recent study, regarding the 3D digital adaptive thorax modelling of people with spinal disabilities, intended for performance-clothing design applications, shows that the developed adaptive thorax model helps to design a basic bodice-pattern design, adapted to the patient’s evolving morphology, by recognizing the anthropometric points from certain parts of the skeleton [30,31].

All the approaches described, of which there are many more, are based on the recognition that static 3D models of the human body are not suitable for the construction of sports, medical and protective clothing. Indeed, clothing that conforms to a static shape can be very uncomfortable when performing daily tasks, such as walking, sitting, or reaching. In studies on virtual prototyping of clothing for people with limited body abilities, design considerations made by using a scanned 3D body model in a sitting posture ensure the ergonomic comfort of the garments when worn in a sitting posture, and take into account the functional requirements imposed by strength and movement limitations, such that they do not lead to additional health problems for paraplegics [32,33].

With the development of computer graphics in the field of human body kinematics and the animation of virtual garments, rigged human-body models also began to emerge, from the very beginning [34,35], to improve deformable virtual humans [36]. For animation purposes, an automatic adaptation of existing general models to scanned 3D body-model mesh was developed to be used for kinematic body models [37]. That there is a growing interest in designing personalized garments in sitting or other postures by using a kinematic 3D body model is reflected in the research [38,39,40,41]. Kozar et al. [38] designed an adaptive 3D body model intended for the development of clothing for people with limited body capabilities using Blender software. The studies by Zhang D. and Krzywinski S. [40,41] highlight that there is currently no kinematic 3D human-body model for representing realistic body deformations during movement, especially in the elbow, knee, and hip joints. Therefore, they investigated four methods, linear-blend skinning in the simulation software Clo3d, auto-rigging of 3D scans on the online service Maximo and a skinned multi-person linear human model and anatomical simulation using the plugin Ziva Dynamics, which also indicates improper deformation of the body mesh during bending of the elbow, knee, and hip joint regions. In recent research by Klepser A. and Pirch K. [9], a new process for generating 3D body models using Blender 3D software outside of 3D garment simulation software was developed, resulting in a parametric and rigged 3D body model that can be used across platforms. From this research, it is evident that, despite carefully performed scanning and mesh processing, difficulties remained due to incorrect mesh deformations during posture adjustments.

Without a realistic 3D human body shape and natural human postures, it is difficult to create properly fitting clothing. In order to achieve a comfortable wear comfort and reduce development time, clothing should be designed based on specific body postures. In a study by Gill and Parker [7], they researched standing scanning postures (scan posture with feet 40 cm apart and a natural, relaxed standing posture with legs closer together) were found to have a significant effect on hip girth and could cause an average change of 2 cm, which can consequently impact garment fit. While moving the body and performing different body postures, body dimensions may change; especially, an increase of lower body girths has been observed in a sitting posture [5,13,19]. Therefore, these changes in body dimensions must also be considered when developing special clothing pattern designs intended for performing different movements or to be worn in specific body postures. In the work of Delph S. L. et al. [42], it is pointed out that the dynamic simulation of movements enables the software that integrates models describing the anatomy and physiology of the elements of the neuromusculoskeletal system and the mechanics of multi-joint movement. Recent trends in 4D-scanning technology predict a major breakthrough in building a kinematic 3D body model that will provide accurate 3D body-mesh deformation and meet the human need for soft-tissue deformations that mirror real humans [43,44]. In the research of Pons-Moll G. et al. [43] two technologies to capture and process the data of full bodies in motion, 4D scanning and 4D-mesh alignment, were used to analyse over 40,000 scans of ten subjects. Analysis of how soft-tissue motion causes mesh triangles to deform relative to a base 3D body model was performed and a Dyna model was created that approximates soft-tissue deformation and relates the subspace coefficients to the changing pose of the body.

The present study focuses on the development of a kinematic 3D human-body model intended for the development of personalized clothes for a sitting posture, using virtual prototyping. A prerequisite for 3D human-body motion is the kinematic structure of a 3D body model, adapted to the anatomical and anthropometric characteristics of the subjects. Therefore, an improved armature in the pelvic region was modelled, using Blender software, and based on a scanned female body in a standing and a sitting posture. Real and virtual measures of female lower-body circumferences for both postures and their changes when moving between postures were investigated to compare changes of the kinematic 3D body model’s mesh during sitting posture adaption. Virtual simulations of personalized- and real-trouser prototypes were investigated to evaluate their fit on standing, sitting and kinematic-sitting 3D body models.

## 2. Methods

### 2.1. Measuring Lower-Body Dimensions in the Standing and Sitting Postures of Real Persons

Measurements of the lower-body dimensions in a standing and a sitting posture were carried out to evaluate changes in lower-body dimensions between the observed postures. A case group of twenty-two female subjects voluntarily participated in the study, ranging in age from 20 to 24 years, with different body heights (BH), body weights (BW) and body mass indexes (BMI). Informed consent was obtained from all subjects involved in the study for the use of body measurements for research. During measurement, participants wore leggings, and measurements were rounded up to the nearest half centimetre. The basic average data of the measured participants are presented in Table 1.

**Figure 1:**
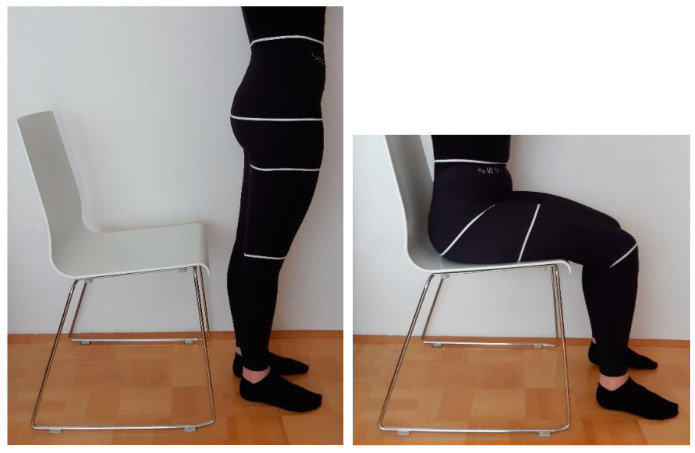
Measurement of real body dimensions.

Examined were those body circumferences that impact the development of clothes for a sitting posture and the lower part of the body (trousers). The four body dimensions, measured according to the standard ISO 8559-1 [45], were: waist circumference, hip circumference, thigh circumference and knee circumference in a standing and sitting posture, as shown in Figure 1. During manual measurements, using a measuring tape, locations of the body dimensions, anthropometric landmarks and the standard procedure for measuring the human body, were considered according to the standard ISO 8559-1 [45]. All lower-limb measurements were taken from the right limbs. In sitting posture, hip circumference was measured transversely as the maximum circumference around the buttocks, while knee circumference was measured at a 90-degree angle with knees bent. Manual measurements were rounded up to the nearest half centimetre.

#### Data Analysis

The mean values ($\bar{x}$), standard deviations (SD) and coefficients of variation (CV) were calculated for the measurements of body dimensions in a standing (MSTA) and measurements in a sitting posture (MSIT). Differences (D) were calculated between the mean values of MSTA and MSIT. A positive value (+) means that the MSTA is greater than the MSIT and a negative value (–) vice versa.

The Pearson correlation coefficient was used to analyse the relationship between basic data of the participants and their body measurements, as well as the relationship between the basic data of the participants and differences in body measurements between the standing and sitting postures. The aim was to evaluate if there were any influences of the basic data of the participants in this case study on twenty-two female subjects’ body height (BH), body weight (BW) and body mass index (BMI) on body measurements for a standing posture and on differences in body measurements between standing and sitting postures (D_MSTA-MSIT_).

### 2.2. Construction of the Kinematic 3D Body Model

The main idea of the kinematic 3D body model is to take a 3D scan, in a standing posture, and to then pose it to a required posture. Since the human body is a complex bundle of bones, muscles, other soft tissues and skin, such a kinematic model must be complex enough to simulate these real structures. Some authors have created virtual models with most of these structures [39]. In this study, scans of the real female body were used, and they were attached to an armature. When building the armature, focus was mainly on the lower parts of the body (the pelvis region, hip joints and knee joints) due to our goal of exploring the sitting posture. To build a correct kinematic 3D body model, two 3D scans of the same person were used, one in a standing and one in a sitting posture. The standing scan was transformed into a kinematic 3D body model by adding an armature that allowed placing the model in a sitting posture, using the sitting scan as a reference.

#### 2.2.1. Three-Dimensional Scanning

To construct the virtual skeleton for the kinematic 3D body model, scanning of female participants was performed in a standard standing posture and in a sitting posture. Informed consent was obtained from the woman who participated in the study.

An ATOS II 400 3D optical scanning system (Gom GmbH, Braunschweig, Germany), with a measurement volume of 1200 mm × 1200 mm, was used for scanning. During scanning, the woman stood and sat with knees bent at 90 degrees and hands slightly raised, breathing normally, and wearing a thin bodysuit. Scanning, with arms raised and with space between the thighs, was carried out for the purpose of the virtual prototyping of clothing. Optical scanning was performed from different angles and heights to digitize a complete person. A real-time surface mesh of the individual scan was acquired using Atos V6.0.2-6 software (Gom GmbH, Braunschweig, Germany). In addition, 3D human-body-mesh modelling techniques and surface-reconstruction techniques, such as manual mesh-holes filling, mesh cleaning and an automatic algorithm called Poisson reconstruction of mesh, which creates a new watertight mesh over the old one, were used to obtain 3D body models in standing and sitting postures. Finally, some manual mesh sculpting and adjustments were performed in critical areas such as the crotch, where the scanner was unable to capture the surface, to fulfil and reliably present the 3D scans, which method is presented in detail in [38].

#### 2.2.2. Armature as a Virtual Skeleton

In this study the open-source creation suite Blender 3D [46,47,48] was used to construct the kinematic 3D human-body model, based on the scanned female body in standing and sitting postures. The whole process was interactive, with much trial and error.

**Figure 2:**
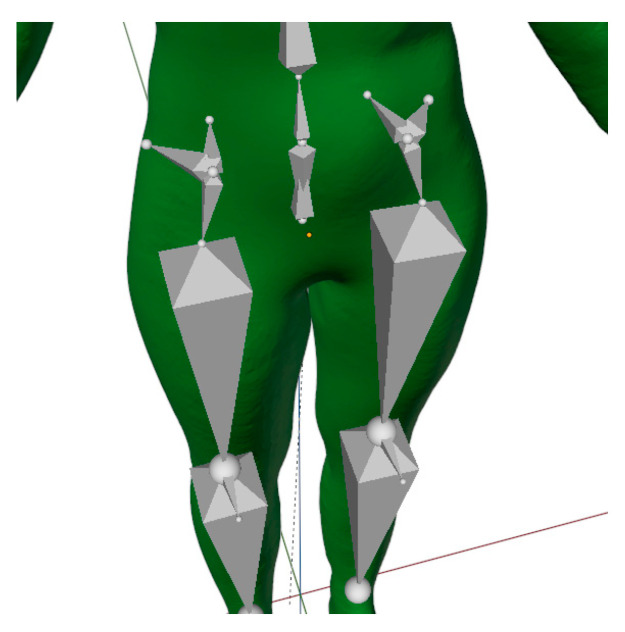
Blender 3D—improved armature of the lower part of the kinematic 3D body model.

**Figure 3:**
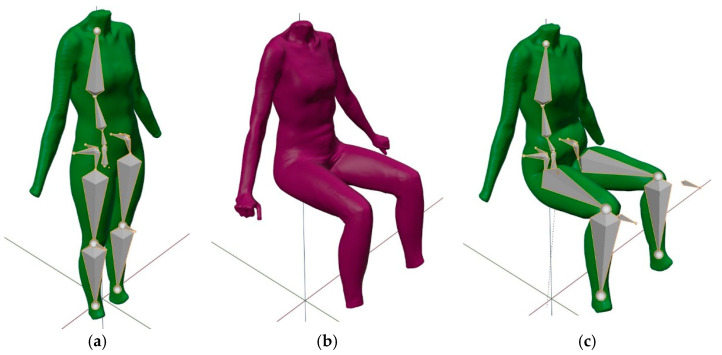
Virtual body models used in this study: (**a**) standing scanned 3D body model with an improved armature, (**b**) sitting scanned 3D body model and (**c**) standing scanned 3D body model, posed with an improved armature, herein called the kinematic sitting 3D body model.

In this study, our focus was on the pelvic region of the torso, as this is a critical region for mesh deformation during sitting-posture adjustment. In the first part of the study, the armature was performed with a simple model consisting of only two bones, one on each side, to connect the spine with the thigh bone. This solution was insufficient because the 3D model deformed inappropriately when changing posture from standing to sitting. Placing the bones of the standing 3D body model in position to achieve sitting position, adequate to scanned body models, lead to missing mesh volume in the buttocks area; moving bones to the back side of the model, to achieve a more fulfilled shape of the buttock, resulted to an overall wrong kinematic model, where too-short thighs appear in the sitting posture. These findings lead to the implementation of two pairs of additional bones, so-called helper bones, in the pelvic region, as shown in Figure 2 and Figure 3.

The built armature’s absolute x, y and z coordinates for each bone’s head and tail in Blender 3D, for a standing posture of a scanned person, are summarized in Table 2, and for a sitting posture in Table 3. Our armature for a sitting posture is based on the reference scan and scanned 3D body model of the sitting posture, which allowed us to accurately compare and evaluate the observed circumferences of the lower-body parts (Figure 1). Blender 3D does not support automatic skeleton adjustment to the 3D body model, therefore manual adjustment was required. The study by [43] made progress in this direction using a 4D scanning technique, in which the additional dimension is time. This means that the change of posture can be accurately examined at each intermediate stage, and that body shape is correctly captured and presented throughout the entire movement sequence.

#### 2.2.3. Rigging

Rigging is a procedure whereby a scanned mesh and armature are bundled into a kinematic 3D body model. The purpose of building a kinematic 3D body model is to adapt the body to the different postures needed for developing personalized garments, and it is performed in a virtual environment, using CAD 3D software (version).

To investigate the kinematic sitting posture, standing and sitting 3D body models of a given participant were used as reference and for comparison (Figure 3a,b). Virtual models were positioned in the space overlapping the upper part of the body. With the kinematic 3D body model (Figure 3a,c; green), we tried to achieve the posture of a scanned sitting 3D body model (Figure 3b; purple).

When building our kinematic 3D body models, we focused on the lower part of the body, containing the hip joints in the pelvic region and the knee joints. For the rigging of the 3D body models’ mesh and armature in Blender 3D, the Parent with Automatic Weights tool was used. The armature enabled us to pose any 3D body-model mesh by selecting the armature and manually moving/rotating the appropriate bones into a desired pose that best fit the sitting 3D body model.

#### 2.2.4. Measuring Scanned 3D Body Models

Measurements of the scanned virtual 3D body models, in standing and sitting postures and in the kinematic sitting 3D body model, were carried out with the aim of exploring whether any changes in the observed circumferences (WC, HC, TC, KC) occurred with the change of the body posture from standing to sitting and between the scanned 3D body model and the kinematic 3D body model in a sitting posture.

All measurements can be referred directly to real body measurements, therefore, the measurement positions are the same as shown in Figure 1. Measurements were performed in Blender 3D using the section plane with the Modifier Boolean Difference tool. Newly created hole circumferences in section planes were selected and evaluated using the Add-in Measure-It tool. All measurements of circumferences were repeated five times. The mean values ($\bar{x}$), standard deviations (SD) and coefficients of variation (CV) were calculated for the measurements of 3D bodies’ dimensions in a scanned standing posture (3DMSTA), scanned sitting posture (3DMSIT) and kinematic sitting posture (K3DMSIT). Differences (D) were calculated between the mean values of 3DMSTA and 3DMSIT, 3DMSTA and K3DMSIT, 3DMSIT and K3DMSIT. A positive value (+) means that the 3DMSTA is greater than the 3DMSIT or K3DMSIT and a negative value (–) vice versa.

### 2.3. Virtual Simulation of Personalized-Trouser and Real-Trouser Prototypes

Construction of the basic trouser-pattern design was performed for the scanned female, whose body height was 165.0 cm and whose virtually measured waist, hip, thigh and knee circumferences from the scanned 3D body model, in standing posture, were recorded (WC = 69.0 cm, HC = 97.5 cm, TC = 54.5 cm, KC = 35.5 cm), using the rules and equations of the construction system M. Müller & Sohn [49] for the calculation of proportional measures (trousers outside length, front and back width, sitting depth, knee height), where the width at the knee line and bottom edge was 20.0 cm. A regular trouser style, made of woven fabric and which fit the body without pressure, was used for this research. Continuous sitting, which is unavoidable for people with immobile lower extremities, the elderly and, often, in many professions, such as drivers, secretaries, machine operators etc., or in continuous work behind a computer for several hours (which we have witnessed rise in the last two years, due to COVID-19), may affect vascular endothelial function in the lower extremities. Indeed, it has been shown that prolonged flexion of the hip and knee joints, as occurs during sitting, and the associated low blood flow caused by arterial bending, is disadvantageous for leg vascular health [50]. In addition, analysis of the effect of different sitting postures on skin temperature of the lower extremity has shown that sitting postures cause a decrease in blood flow volume to the lower extremities, resulting in a decrease in lower-extremity temperature. Especially, sitting with the legs crossed affects the circulation of blood-flow volume much more than normal sitting with uncrossed legs, on a chair [51].

In order to investigate the influence of the body-posture change from standing to sitting on changes in the lower-body dimensions, and thus on the fit of trousers to the body in different postures, the first basic trouser-pattern design was constructed for the standing posture, without ease allowances in waist and hip circumferences, and the second basic trouser-pattern design was constructed with minimal ease allowances, based on investigated differences of circumferences between the mean values of the MSTA and MSIT. Since the dimensions of the thigh circumference and knee circumference are smaller than the dimensions in the regular basic trouser-pattern design, we did not add any ease allowance in these construction lines.

The construction of the personalized trouser-pattern designs was performed using the OptiTex PDS system. Three-dimensional simulations of the trouser prototypes and analysis of their fit on the standing, sitting and kinematic sitting 3D body models were performed using the 3D and Tension modules of the OptiTex PDS V11 system. A 3D Tension Map and the Tension XY tool were used to measure the highest tension of the fabric of the trousers in both the warp (X) and weft (Y) direction, and together (XY), which are also presented individually for the back part. Fit evaluation, based on the visual assessment of colour maps, can be subjective, because of small, nuanced differences in colour, and because colour maps represent the maximum value in red and the minimum value in blue for each garment, regardless of absolute values [52,53,54]. Some researchers [54,55,56,57] have included the numerical values provided by colour maps in their analyses, since the tension maps of two different fabrics may look similar while their maximum tension values may differ. The amount of virtual tension influencing the fabric is given by the OptiTex PDS V11 system in units of gf/cm (i.e., forces acting in a unit of length), which has here been converted into SI units N/cm. The colour band on the tension scale ranges from red to yellow, through green, to blue, where red indicates the maximum values and blue the minimum values of virtual tension. By default, the Tension Map module displays the highest value of tension found in the garment.

A 100% cotton plain-weave fabric with a surface mass of 154.94 gm^−2^ was used for the trouser simulations and the sewing of real-trouser prototypes for a comparison of their fitting. The linear density for the warp was 32 tex and, for the weft, was 38 tex. The fabric density in the warp direction was 25 threads/cm and, in the weft direction, was 22 threads/cm.

The low-stress mechanical parameters of the used fabric (extensibility, bending rigidity, shear rigidity, surface thickness) were measured using the FAST measuring system, Table 4, and considered for trouser virtual simulations by using Fabric Converter of OptiTex software, which converts extensibility (%) in resistance to stretch (gf/cm), bending rigidity (μNm) in resistance to bend (dyn·cm), shear rigidity (N/m) in resistance to shear (dyn·cm), thickness (cm) and surface mass (gm^−2^).

## 3. Results with Discussion

### 3.1. Comparison of Real Human Body Circumferences between Standing and Sitting Postures

The mean values ($\bar{x}$), Standard deviations (SD) and Coefficients of variation (CV) for both the measurements in a standing posture (MSTA) and measurements in a sitting posture (MSIT) of the real female bodies are presented in Table 5. In addition, the minimum and maximum values of each body dimension, the calculated differences (D) between the mean values of the MSTA and MSIT (D_MSTA-MSIT_) and the percentages of these differences are given in Table 5.

Based on the measured body dimensions, it was found that changes occur in all measured circumferences when subjects change their posture from standing to sitting. The average waist circumference (WC), which was 77.25 cm in the standing position, increased to 77.98 cm or an average of 0.73 cm (0.94%). In the sitting posture, the hip circumference (HC) also increased by an average of 3.23 cm (3.03%), thigh circumference (TC) by an average of 0.82 cm (1.32%) and knee circumference (KC) by an average of 1.48 cm (3.71%), Table 5.

**Figure 4:**
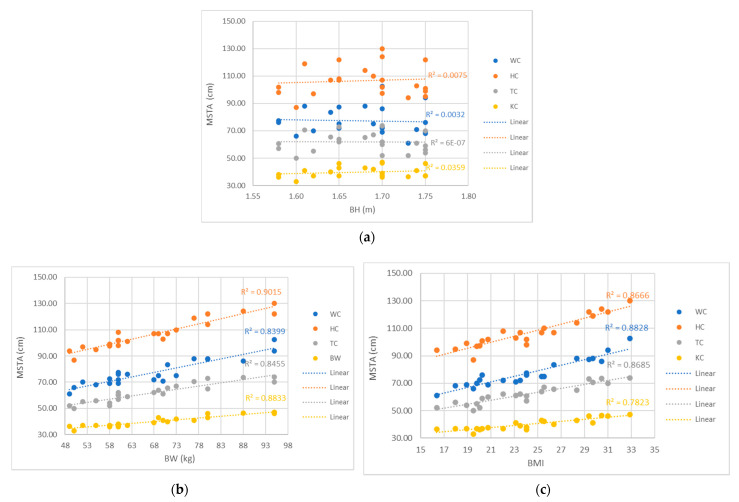
Influence of participants’ basic data on observed measurements of body circumferences in the standing posture (MSTA): (**a**) body height, (**b**) body weight, (**c**) body mass index.

**Figure 5:**
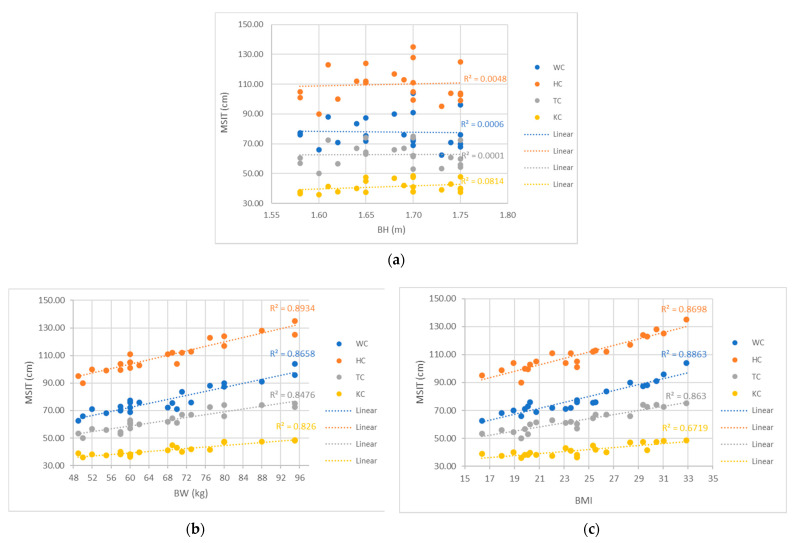
Influence of participants’ basic data on observed measurements of body circumferences in a sitting posture (MSIT): (**a**) body height, (**b**) body weight, (**c**) body mass index.

The analyses of measured circumferences show that, with increasing body weight (BW) and body mass index (BMI), WC, HC, TC and KC increase, while no effect of body height (BH) on the measured circumferences was been observed for a standing (Figure 4) or sitting (Figure 5) posture.

High linear trends of the increase in the measured circumferences of the tested participants with the increase in their BW (R^2^ between 0.8399 to 0.9015 for MSTA and between 0.8260 to 0.8934 for MIST) and BMI (R^2^ between 0.7823 to 0.8828 for MSTA and between 0.6719 to 0.8863 for MIST), were observed for both the seated and standing postures (Figure 4 and Figure 5). The greatest correlation was found between the increase in hip circumference with increasing body weight and BMI. Measuring the hip circumference in the construction of trouser-pattern design is extremely important for worn comfort, especially when sitting.

In Figure 4 and Figure 5, more than half of the study participants had a normal BMI, i.e., between 18.5 and 24.9 (50.00%), BMI below 18.5 or underweight had only 9.09%, pre-obesity 27.27% and obesity class I 13.64%, the categories of which are addressed in the source [58] for adults over 20 years.

In this case study of twenty-two female subjects aged between 20 and 24 years, a wide range of tested participants was measured, with respect to their physical characteristics, as shown in Table 1 and Figure 4 and Figure 5. Therefore, the relationships between the basic data of participants (BH, BW, BMI) and body measurements (WC, HC, TC, KC) were examined using Pearson correlation coefficients (Table 6).

The Pearson correlation coefficients clearly show and support the above findings that all measured body circumferences are influenced by BMI, which is influenced to a greater extent by body weight compared with body height. The Pearson correlation coefficients indicated a very high and strong association of BW and BMI with all measured body circumferences, Table 6.

No obvious trends were found between the basic data of the participants studied and the calculated differences in the measured circumferences. The Pearson correlation coefficients also show a slight-to-weak association of the studied data and relationships between the basic data of the participants and the differences (D) of the body measurements between the standing and sitting postures (DWC_MSTA-MSIT_, DHC_MSTA-MSIT_, DTC_MSTA-MSIT_, DKC_MSTA-MSIT_), Table 7.

It was found that the average increases in measured circumferences in the sitting posture (WC, HC, TC and KC) did not depend on BH, BW or BMI. Therefore, it can be concluded that there is a difference in the measured circumferences when the posture is changed from standing to sitting, due to the change in the shape of the joints and the redistribution of the muscles and adipose tissue surrounding the joint, resulting in an increase of the observed circumferences.

Based on the comparison of real female-body circumferences between standing and sitting postures, it can be concluded that the measured body circumferences are mainly influenced by body weight, regardless of body height. Moreover, when changing postures from standing to sitting, the measured body circumferences increased on average regardless of body height: WC = 0.73 cm, HC = 3.23 cm, TC = 0.82 cm, and KC = 1.48 cm. Thus, it can be pointed out that, in the process of construction of basic clothing-pattern designs, intended for garments of a woven fabric, ease allowances must be added to the body dimensions to ensure the unhindered movement of the person in the garment. When determining clothing dimensions, body dimensions are constant and ease allowances variable, which is discussed in detail in the paper by [59,60]. The correct amount of ease allowance provides a well-fitting and functional garment. Generally, ease allowance is divided into two types [61]. First, the wearing ease allowance, which refers to the amount of added fabric allowed over and above the body dimensions to ensure the comfort, mobility and drape of clothing, and secondly, design ease allowance, which refers to the amount of added fabric to achieve a particular design and shape. Ng R. et al. [62] classified ease allowances according to three different functions: (a) basic movement, breathing and sitting, which require static ease allowances and also known as standard ease; (b) nonstandard body shapes (fat, thin, big hip, strong leg,…) and their movements (walking, jumping, running, etc.), which require dynamic ease allowances; and (c) styling ease, which is the extra spacing required to conform the desired shape. Chen Y. et al. [63] also point out importance of the fabric ease allowance, which considers the influence of the mechanical properties of clothing fabric on the positive or negative ease allowance. The properties of stretchy knitted fabrics require special garment-pattern design and negative ease allowance.

When constructing basic pattern designs for regular trousers intended for woven fabric, standard ease allowances are usually added to the waist and hip circumference, while the circumference of the trouser leg is adjusted at the thighs and knees, according to the clothing’s model. In the study by McKinney et al. [64], the body-to-pattern measurement and shape relationships in trouser patterns, drafted by two methods, were explored. It was found that their body-to-pattern measurement and shape relationships were inconsistent between and within the methods, making them unsuitable for use in computer-aided custom patternmaking. A comparative study of four trouser-pattern-making methods (Aldrich, Armstrong, Bunka, and ESMOD) was carried out on a standard standing and five dynamic postures of a female subject, aged 29, and with a BH of 167.7 cm, BW of 68 kg and BMI of 24.1 [65]. All pattern-making methods provided different amounts of ease allowances for the waist and hip girths, where the Armstrong method added the greatest amount (8.88 cm in the waist and 6.34 cm in the hips), while other methods suggested at least 3.0 cm in the waist and 4.0 cm at the hips. All pattern-making methods provided construction of trousers for the woven fabric, where slight differences between construction methods and the suggested amounts of ease allowances influenced the clothing’s fit and overall shape. Specifically, dissatisfaction increased slightly when the subject changed poses, and their discomfort increased when changing postures (stepping at walking pace, sitting 90˚, stooping 90˚, climbing the stairs, squatting on hams). Studies on the fit of women’s trousers by measuring the differences between the trousers and body scans (ease) for twenty-four subjects, aged 35 to 55 years, representing three body-shape groups, showed no clear dependence on body shape [66]. Research on trouser fit was conducted for women aged 55 years and older and tested 176 participants in five states, the results of which highlighted that variations in body size, shape, proportion, and posture create difficulties for effective ready-to-wear sizing systems with a practical number of sizes [67]. Therefore, for a ready-to-wear sizing system, a comprehensive measurement of the population has been carried out in last decades, using 3D human-body scanning [1,6]. In the research by Petrak S. and Mahnić Naglić M. [5], two dynamic body postures (standing, sitting) were investigated in 80 female subjects, aged 20 to 30 years. It was found that with a change of the body posture from standing to sitting, hip width increased in the range of 0.7 to 6.0 cm, and it can be assumed that hip girths also increased. Similarly, it was also found in case studies on the development of personalized clothing in smaller number of female and male test subjects that, with a change of the body posture from standing to sitting, hip girth increased [13,32].

Based on the results of the case studies of women described in this section, it can be assumed that when constructing basic pattern-designs for regular female trousers intended for woven fabric, it is necessary to add a minimal ease allowances on measures of the waist circumference and hip circumference of WC = 0.73 cm, HC = 3.23 cm, and for trousers with narrower trouser legs, ease allowances on the thigh and knee circumference of minima for TC = 0.82 cm and KC = 1.48 cm.

### 3.2. Virtual 3D Body Models in Sitting Posture

Creating a kinematic 3D body model requires much skill and knowledge in and of 3D modelling software. Most methods are manual, and there is usually a lot of trial and error.

**Figure 6:**
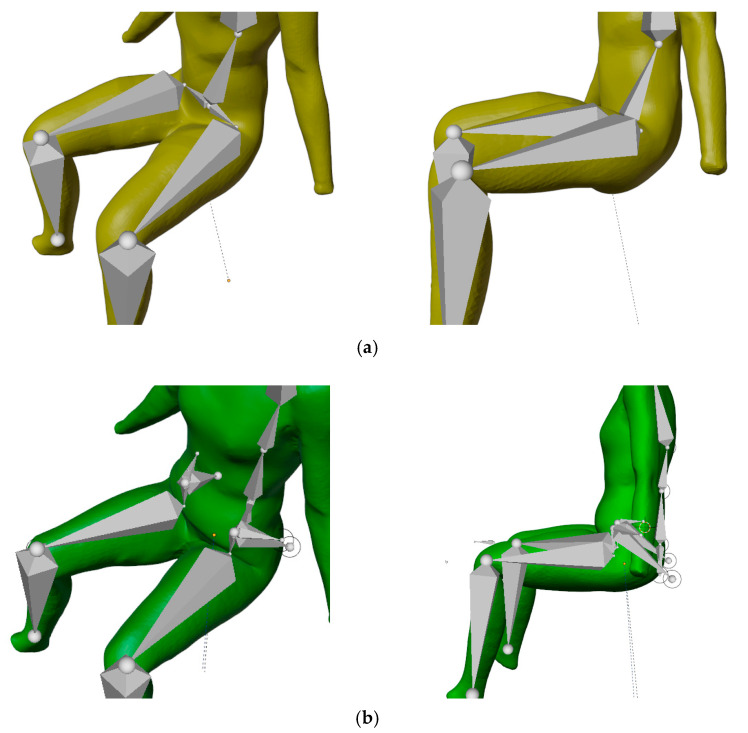
Armature structure of kinematic 3D body model: (**a**) simple armature in pelvic region, (**b**) improved armature with helper bones in pelvic region.

A simple armature in the pelvic region [38,39,40,41,68,69] was used in the preliminary study, which produced too-short legs and thus incorrect knee position. If the femur bone was moved, the buttocks were unnaturally curved, with a lack of volume, as shown in Figure 6. Subsequently, we introduced and tested additional bones in the pelvic area. With the implementation of so-called helper bones, the kinematic 3D body model retained the appropriate kinematics of the tibia, femur, and retained the shape of the buttocks and thighs in the folded area of a sitting posture. In Figure 6b, an improved armature structure with helper bones in the pelvic region is shown, which enabled more suitable kinematics for moving between the standing and sitting postures of the kinematic 3D body model, which featured corrected positions for the knees and buttocks, as well as buttock shape.

**Figure 7:**
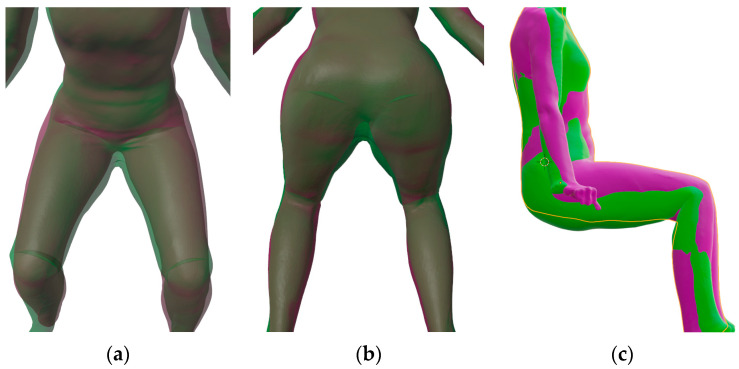
Overlapping meshes of a sitting 3D body model (3DBMSIT) (purple) and kinematic sitting 3D body model (K3DBMSIT) (green): (**a**) the upper front view, (**b**) the lower bottom view, (**c**) side view.

When analysing 3D body models in sitting posture, there were some shape differences between the scanned sitting 3D body mesh (purple) and kinematic sitting 3D body mesh (green) in Figure 7. Due to transparent representation of the scanned sitting 3D body mesh and kinematic sitting 3D body mesh in Figure 7a,b, green areas at the crotch and, on the other side, purple areas at hips, are noticeable.

The smaller crotch in the kinematic sitting 3D body mesh can be explained by mesh filling and repairing in the scan-preparation phase. When 3D scanning the human body, there are some places that are difficult to capture in a standing posture, such as the crotch or the armpits, where the scanner cannot capture the surface. Holes and gaps appear in the 3D scan, and they must be filled for a correct virtual garment pattern design. This reconstruction can also produce an inaccurate mesh that is visible in a sitting posture. Wider hips on the scanned 3D sitting mesh can be interpreted as the fact that in a real human body in sitting posture, the soft tissues in the sitting area are pressed and stretched around the hips as shown in Figure 7c.

**Figure 8:**
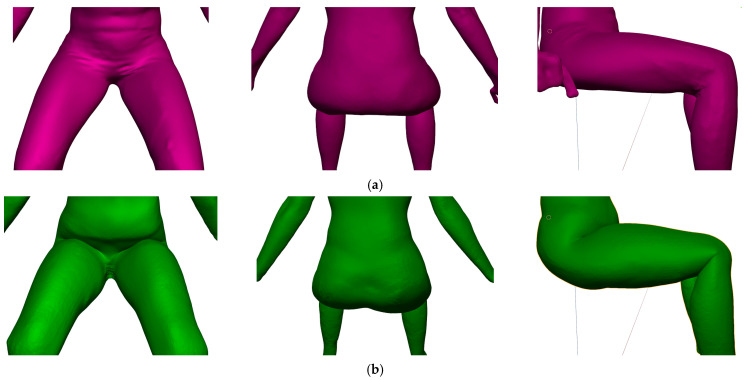
Comparison of joints regions: (**a**) scanned sitting 3D body model (purple), (**b**) kinematic sitting 3D body model (green).

The standing posture requires muscular activity and tension, therefore the geometry of the body is different in the standing and sitting postures. These differences can be seen very well in Figure 8 and will be evaluated in Section 3.2.1 with measured circumferences of the lower body parts. Even with the improved armature in the pelvic region, differences between the scanned and kinematic sitting 3D body models in the flexion of the hip joints in the pelvis, which unnaturally moves the soft tissues towards the abdomen, can be clearly seen in Figure 8. A pronounced unnatural fold under the bended knees can be also seen. In general, the real human body is not a uniform solid model, but is built of different tissues and structures. For this reason, building a kinematic 3D body model that will simulate real-body behaviour becomes more challenging. Researchers [9,39,40,41] have had the same issues of inappropriate 3D body mesh bending in regions, where the bending angle is large.

There are some services already available, wherein virtual characters can be downloaded, but they are intended for animation [40]. These characters consist of a 3D body mesh and a simple inner armature, suitable for animation. Additionally, for 3D animation the inappropriate 3D body-mesh bending can be hidden with some additional geometry or post-processing effects, but for clothing-pattern design, a clean and correct geometry is required. For this purpose, an improved armature in pelvic region and additional helper bones for proper flexion of hips joints in pelvis, respectively, were added to the armature of the standing kinematic 3D body model in this study, to simulate the posture, size, and shape of kinematic model as closely as possible to the scanned sitting 3D body model.

The current solution for 3D body models intended to simulate clothing is to manually adjust and reconstruct the mesh. However, these adjustments are not possible without a 3D scan of the intended wearer in a sitting posture, so some predictions and guidelines are suitable at this point. In future research, incorporating as many simulated tissues as possible is likely to be the preferred method for creating kinematic 3D body models for virtual clothing-pattern design. Some available solutions are intended for modelling, simulation, and analysis of the neuromusculoskeletal system of the human body as used in [42,43].

#### 3.2.1. Comparison of Scanned 3D Body-Models’ Measurements between the Standing (3DMSTA) and Sitting (3DMSIT) Postures and the Kinematic 3D Body Model in Sitting Posture (K3DMSIT)

The mean values ($\bar{x}$), standard deviations (SD) and coefficients of variation (CV) were calculated for measurements of 3D body dimensions in scanned standing posture (3DMSTA), scanned sitting posture (3DMSIT) and kinematic sitting posture (K3DMSIT), Table 8. The mean values of circumferences are rounded up to the nearest half centimetre. Differences (D) were calculated between the mean values of 3DMSTA and 3DMSIT, 3DMSTA and K3DMSIT, 3DMSIT and K3DMSIT, Table 9. A positive value (+) means that the 3DMSTA is greater than the 3DMSIT or K3DMSIT and a negative value (–) vice versa.

Standing postures require muscle activity and tension, therefore the observed circumferences differ between scanned 3D standing body model, scanned 3D sitting body model and kinematic 3D sitting model. The analysis of the measurement results of the 3D body models shows circumferential differences when changing posture from standing to sitting, with respect to the real person’s measurements. In sitting postures, all measured circumferences show an increased value. The highest increase was seen in the waist and knee circumferences, which is consistent with the results of the real measurements in Table 5 and Table 9. The waist and hip circumferences of the kinematic sitting 3D body model decreased, as compared with the standing and sitting 3D body models, while the circumferences of the thigh and knee.

The differences between the mean values of the standing and sitting 3D body models (D_3DMSTA-3DMSIT_) showed an increase in circumferences, which were for WC of 2.20 cm (3.20%), HC of 5.40 cm (5.54%) TC of 2.80 cm (5.14%) and KC of 3.60 cm (10.17%) (Table 9). The differences between the mean values of the standing 3D body model and kinematic sitting 3D model (D_3DMSTA-K3DMSIT_) indicated a decrease in the waist and hips circumferences of the kinematic sitting 3D body model, while its thigh and knee circumferences increased. Significant differences between mean values are observed between the two sitting 3D body models (D_3DMSIT-K3DMSIT_) (Table 9). Especially the hip circumference of the kinematic sitting 3D body model is 6.8 cm smaller than that of the sitting 3D body model, which can also be observed in Figure 8. The large HC difference can be explained by the lack of 3D program tissue simulation ability, while in Blender 3D, automatic weights were used for the armature—mesh interference. With some mesh weights, better adaptation and redistribution results can be expected. A large, triangular 3D scan was used for the kinematic 3D body model in this case, moreover, researchers in [40] used the quadratic simplified 3D mesh model, Skinned Multi-Person Linear (SMPL), which promises easier handling, but it still has a lack of proper bending at joints with large angular rotations.

### 3.3. Virtual Simulations of Personalized Trousers on Standing, Sitting and Kinematic Sitting 3D Body Models and Real-Trouser Prototypes

**Figure 9:**
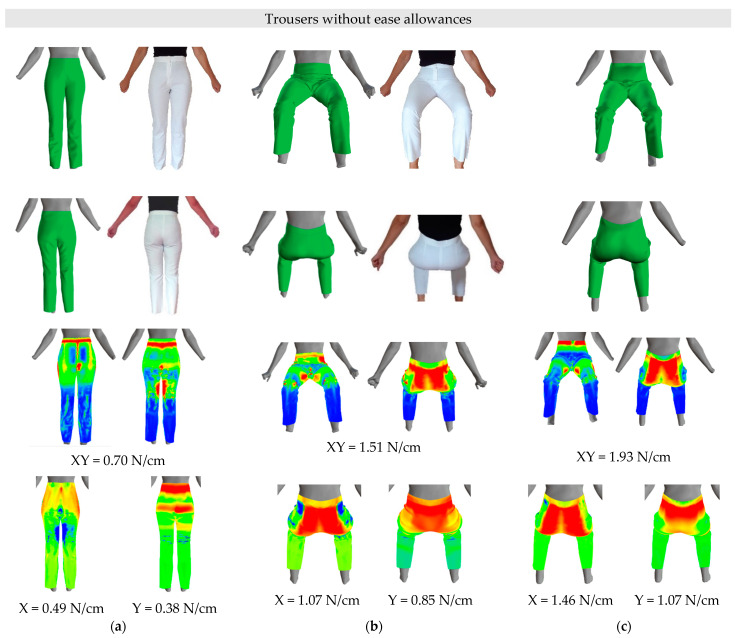
Virtual and real-trouser prototypes without ease allowances and tension in trouser fabric: (**a**) standing 3D body model and real person, (**b**) sitting 3D body model and real person, (**c**) kinematic sitting 3D body model.

**Figure 10:**
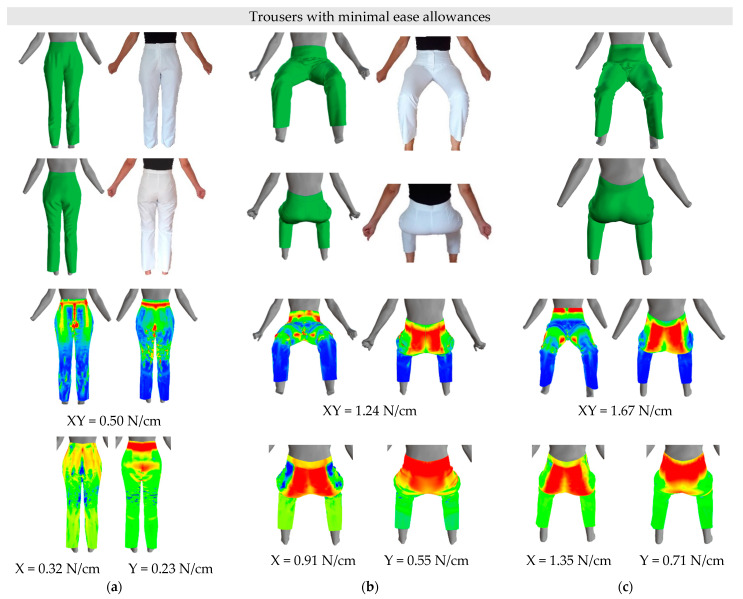
Virtual and real-trouser prototypes with minimal ease allowances and tension in trouser fabric: (**a**) standing 3D body model and real person, (**b**) sitting 3D body model and real person, (**c**) kinematic sitting 3D body model.

Virtual simulations of the basic trouser-pattern design were carried out for a female participant with a body height of 165.0 cm, and her virtually measured waist, hip, thigh, and knee circumferences on the scanned 3D body model in a standing posture were: WC = 69.0 cm, HC = 97.5 cm, TC = 54.5 cm and KC = 35.5 cm. The real-trouser prototypes were sewn for the same person. The first pair of trousers were constructed without ease allowances (Figure 9) and the second pair of trousers with minimal ease allowances (Figure 10). The researched average differences in circumferences between standing and sitting postures (Table 5) were rounded up to the nearest half centimetre and used as standard ease allowances. Therefore, the ease allowances were 1.0 cm for the waist circumference and 3.5 cm for the hip circumference. The thigh and knee circumferences were not considered, because regular-fit trousers have a larger circumference of the trouser leg than the measured thigh and knee circumferences, even if we add ease allowances for the TC of 1.0 cm and for the knee KC of 1.5 cm. For the virtual simulation used 100% cotton woven fabric, had low extensibility in the warp direction (0.93%) and high shear rigidity (189.23 Nm^−1^), while its extensibility in the weft direction was 5.6%, and bending rigidity of 19.08 μNm in the warp direction and 7.46 μNm in the weft direction.

Different fits of the virtual trouser prototypes, with and without minimal ease allowances, were between different 3D body models. A perfect fit of the trousers without ease allowances is observed on the standing 3D body model (Figure 9a), while, considering minimal ease allowances, a slightly wider virtual prototype of the trousers can be seen (Figure 10a). The latter is also reflected in the tension of the fabric used for the trousers in this study. In a standing posture, the maximum tension XY in trousers without ease allowances was 0.70 N/cm (X = 0.49 N/cm, Y = 0.38 N/cm) and for trousers with minimal ease allowances it decreased to 0.50 N/cm (X = 0.32 N/cm, Y = 0.23 N/cm). Tension in the fabric was observed mainly in the waist region and at the hips and knees (red), which decreased when simulating trousers with minimal ease allowances around the waist and at the hips and knees. The latter can also be seen in the fit of the real pants to the participant. When observing the maximum tension in the warp and weft directions, a decrease in maximum tensions were found for trousers with minimal ease allowance, and the colour maps thereof showed a decrease in tension, especially of the hip-girth region in the weft direction.

On the sitting 3D body model, the trousers with and without minimal ease allowances in the buttocks region were quite tight (Figure 9b and Figure 10b), which is consistent with an increase of the measured real and virtual circumferences in the sitting posture (Table 5 and Table 8) and reflects an increase of the tension in virtual and real trousers. The maximum tension, XY, in the trouser fabric was higher in a sitting posture, compared with a standing posture, and applies for trousers without ease allowances 1.51 N/cm (X = 1.07 N/cm, Y = 0.85 N/cm), while for trousers with minimal ease allowances, it decreased to 1.24 N/cm (X = 0.91 N/cm, Y = 0.55 N/cm) for a sitting posture. The same can be seen for the real trousers, where a small fold of fabric appears in the region between the waist and hips, especially on the right side. The highest tension was observed around the buttocks (red), which, on the one hand, is attributable to the increase in circumference around the waist and hips, and, on the other hand, to the regular trouser-pattern design, which is intended for a standing posture. The decrease in virtual tension of the fabric on the colour map is more difficult to detect. The increase in virtual fabric tension can also be attributed to low fabric extensibility in the warp direction and high shear rigidity (Table 4), which is exacerbated by a sitting posture with restraint of the trousers at the waist and buttocks. In the sitting posture, the trousers’ height at the back area is too low, therefore, back trouser-pattern pieces should be raised to the waistline to reduce the trousers’ tension in the back area and to increase comfort when sitting in them. It should be noted that it is extremely difficult to photograph virtual and real-trouser prototypes from the same angle. Therefore, the body postures and details of fitting the trousers to the test subject and her 3D body model may differ slightly.

Sitting 3D body models differ in measured circumferences and in the shape of the buttocks and abdomen. Although the circumferences around the waist and hips of the kinematic sitting 3D body model are smaller, Figure 9c and Figure 10c compared to these circumferences of the sitting 3D body model, Figure 9b and Figure 10b, the tension in these trousers increased. This is attributed to the bending of the kinematic 3D body model from a standing to a sitting posture in the pelvic region and unrealistic tissue redistributions around the waist and hips. For a kinematic sitting 3D body model, the maximum tension (XY) in trousers without ease allowances was 1.93 N/cm (X = 1.46 N/cm, Y = 1.07 N/cm) and, for trousers with minimal ease allowances, decreased to 1.67 N/cm (X = 1.36 N/cm, Y = 0.71 N/cm), which is consistent with a decrease in virtual tension of the trousers with minimal ease allowance on a sitting 3D body model. This decrease in the virtual tension of the fabric on the colour map is more difficult to detect.

The findings of this study agree with those of a study wherein the influence of different ease allowances on the virtual fit of basic bodice-pattern designs was investigated [57]. It was found that, as the ease allowance increases (0/3/6/9/12 cm), the maximum virtual tension in the woven fabric decreases, from a value of 0.61 N/cm for a zero ease allowance to 0.15 N/cm for an ease allowance of 12 cm.

Based on the results in this part of our research, we conclude that, with the virtual simulation of trousers and real-trouser prototypes designed according to the dimensions of a standing person, it is necessary to consider at least minimal ease allowances on trouser-construction lines for comfort while sitting. On the other hand, we have found that our kinematic sitting 3D body model is not yet reliable enough to develop personalized clothing pattern designs, since, so far, the 3D body mesh does not follow real changes in circumferences and shapes in a sitting posture, despite a carefully planned anatomical skeleton.

## 4. Conclusions

Virtual prototyping of personalized clothing has been a current and reliable tool for some time. Typically, this involves the use of scanned 3D body models, in both standard and specific postures, that are important for the development of personalized clothing. By building a reliable kinematic 3D body model, we could shorten, for the needs of personalized clothing development, the usual process of scanning the body in different postures as a process of fitting and prototyping of real personalized clothing.

This study focused on the development of a kinematic 3D human-body model intended for a sitting posture by using Blender software. It was based on a scanned female body in standing and sitting postures. In the construction of the armature, the focus was mainly on the lower body parts of the pelvic region, hip, and knee joints, due to our exploration of the sitting posture. The study’s participants were female, real and virtual measures of lower-body circumferences in both postures. Virtual prototyping of trousers and sewing of real-trouser prototypes were performed to investigate their fit and comfort on scanned and kinematic 3D body models, as well as on a real body. Namely, to examine prolonged flexion of the hip and knee joints that occurs during sitting and the associated low blood flow caused by arterial flexion, as such are unfavourable for leg vascular health.

It was found that by changing the body posture from standing to sitting, the real and virtual body circumferences changed, which affected the fit and comfort of the virtual and real trousers. Our analysis showed that the measured circumference of the lower body parts increases with body weight and BMI, while an effect on circumferential difference when changing posture from a standing to a sitting was not observed. Therefore, it could be supposed that in a sitting posture, the increase in circumferences of the lower body parts reflects, on average, all tested persons regardless of their basic physical characteristics. The findings on the case group in this research highlight that research is needed for the production of ready-to-wear clothing, as are anthropometric surveys to determine the ease allowances for various construction systems, body postures and textile materials. An increase in measured circumferences was found also for the sitting 3D body model and is attributed to the redistribution of body muscles and adipose tissue surrounding the joints, as well as changes in joint shapes in the regions of body flexion, which are not represented equally on the kinematic sitting 3D body model, regardless of our improved armature in the pelvic region. This part of the research also provides guidelines for software developers and for upgrading kinematic 3D-body-model-mesh deformation when changing body postures.

Based on this study’s measured results, it can be concluded that average increases in waist, hip, thigh, and knee circumferences should be included in the process of designing basic female clothing patterns for a sitting posture, as minimal ease allowances when using a woven fabric, and, in the future, armatures should be constructed with muscles and adipose tissues to achieve realistic circumferences and shapes at the observed regions of a kinematic 3D body model in a sitting posture.

## Figures and Tables

**Table 1:** Basic data of the measured participants.

Age and Body Attributes	Symbol	$\bar{x}$ (x_min_; x_max_)	SD (cm)	CV (%)
Age (years)	A	21.82 (20; 24)	1.26	5.77
Body height (cm)	BH	168.00 (158.00; 175.00)	0.06	3.36
Body weight (kg)	BW	67.73 (49.00; 95.00)	13.57	20.03
Body mass index	BMI	24.07 (16.37; 32.87)	4.69	19.50

**Table 2:** Armature’s absolute x, y and z coordinates for every bone’s head and tail in Blender 3D, for the standing posture of a scanned person.

Bone Part Standing	Bone Head Location			Bone Tail Location			
Bone Name	x	y	z	x	y	z	Parent Bone
Bone.002	−6.25	61.55	1107.00	−4.64	26.30	1333.00	Bone.003
Bone.003	−7.33	27.08	978.80	−6.25	61.55	1107.00	Bone.001
Bone.001	−6.81	48.95	817.00	−7.33	27.08	978.80	Pelvis Master
Pelvis Master	−6.81	48.97	722.40	−6.81	48.95	817.00	-
Pelvis Son	−6.81	48.95	817.00	−6.81	48.97	722.40	Pelvis Master
Leg L Base	79.78	−87.49	872.89	80.15	−86.11	791.70	Pelvis Son
Leg L	80.15	−86.11	791.70	86.63	−59.00	420.60	Leg L Base
Leg L.001	86.63	−59.00	420.60	77.81	−33.04	30.17	Leg L
Buttocks L	79.77	−87.49	872.89	89.68	72.03	720.10	Leg L Base
Buttocks L.001	79.77	−87.49	872.89	137.90	1.46	835.90	Buttocks L
Leg R Base	−99.35	−87.49	871.29	−114.93	−87.49	792.91	Pelvis Son
Leg R	−114.93	−87.49	792.91	−106.22	−59.00	419.18	Leg R Base
Leg R.001	−106.22	−59.00	419.18	−82.70	−33.04	31.69	Buttocks R
Buttocks R	−99.35	−87.49	871.29	−108.00	71.90	719.70	Leg R Base
Buttocks R.001	−99.35	−87.49	871.29	−145.33	1.80	854.00	Buttocks R

**Table 3:** Armature’s absolute x, y and z coordinates for every bone’s head and tail in Blender 3D, for the sitting posture of a sitting posture.

Bone Part Sitting	Bone Head Location			Bone Tail Location			
Bone Name	x	y	z	x	y	z	Parent Bone
Bone.002	−6.87	67.53	1112.00	−3.75	22.40	1336.00	Bone.003
Bone.003	−7.42	51.64	980.30	−6.87	67.53	1112.00	Bone.001
Bone.001	−6.81	48.97	817.00	−7.42	51.64	980.30	Pelvis Master
Pelvis Master	−6.81	48.95	722.40	−6.81	48.97	817.00	-
Pelvis Son	−6.81	48.97	817.00	−6.81	48.95	722.40	Pelvis Master
Leg L Base	79.78	−87.50	872.90	80.15	−86.11	791.70	Pelvis Son
Leg L	80.15	−86.11	791.70	179.80	−446.10	783.40	Leg L Base
Leg L.001	179.80	−446.10	783.40	206.70	−482.80	396.10	Leg L
Buttocks L	79.78	−87.50	872.90	117.50	81.44	735.40	Leg L Base
Buttocks L.001	79.78	−87.50	872.90	151.00	−7.02	862.90	Buttocks L
Leg R Base	−99.34	−87.50	871.30	−94.96	−85.95	791.60	Pelvis Son
Leg R	−94.96	−85.95	791.60	−187.30	−447.90	783.30	Leg R Base
Leg R.001	−187.30	−447.90	783.30	−202.10	−485.20	394.70	Buttocks R
Buttocks R	−99.34	−87.50	871.30	−158.80	80.16	741.80	Leg R Base
Buttocks R.001	−99.34	−87.50	871.30	−158.80	−4.57	872.80	Buttocks R

**Table 4:** Mechanical parameters of the used fabric measured by FAST measuring system.

Mechanical Parameters	Direction	Unit	Measured Value
Extensibility (E100)	warp	%	0.93
	weft	%	5.60
Bending rigidity (B)	warp	μNm	19.08
	weft	μNm	7.46
Shear rigidity (G)	-	Nm^−1^	189.23
Surface thickness (ST)	-	mm	0.151
Surface mass (W)	-	gm^−2^	154.94

**Table 5:** Body measurements in standing and sitting postures and the differences between the mean values of the MSTA and MSIT.

Body Dimensions	Symbol	MSTA			MSIT			D_MSTA-MSIT_	
		$\bar{x}$ (cm) $x_{\min}$ (cm) $x_{\max}$ (cm)	SD (cm)	CV (%)	$\bar{x}$ (cm) $x_{\min}$ (cm) $x_{\max}$ (cm)	SD (cm)	CV (%)	$\bar{Dx}$ (cm)	$\bar{Dx}$ (%)
Waist circumference	WC	77.25 61.00 102.50	10.15	13.14	77.98 62.50 104.00	10.64	13.64	−0.73	−0.94 ^1^
Hip circumference	HC	106.61 87.00 130.00	11.17	10.47	109.84 90.00 135.00	11.58	10.54	−3.23	−3.03
Thigh circumference	TC	61.95 50.00 74.00	7.30	11.78	62.77 50.00 75.00	7.53	11.99	−0.82	−1.32
Knee circumference	KC	39.84 33.00 47.00	4.00	10.04	41.32 36.00 48.50	4.13	10.00	−1.48	−3.71

^1^ Note: the minus sign means that the body dimension increased when the body changed its posture from standing to sitting.

**Table 6:** Pearson correlation coefficients between participants’ basic data and their body measurements.

Basic Data/Body Measurements	WC	HC	TC	KC
BH	−0.057	0.086	−0.001	0.189
BW	0.916	0.949	0.920	0.940
BMI	0.940	0.931	0.932	0.884

**Table 7:** Pearson correlation coefficients between the basic data of the participants and differences of body measurements between the standing and sitting posture.

Basic Data/Body Measurements	DWC_STA-SIT_	DHC_STA-SIT_	DTC_STA-SIT_	DKC_STA-SIT_
BH	−0.287	0.135	0.118	−0.377
BW	−0.501	−0.286	−0.301	0.004
BMI	−0.401	−0.340	−0.264	0.135

**Table 8:** Three-dimensional body measurements of standing (3DMSTA), sitting (3DMSIT) and kinematic sitting (K3DMSIT) postures.

Measurements of 3D Body Models	Symbol	3DMSTA			3DMSIT			K3DMSIT		
		$\bar{x}$ (cm)	SD (cm)	CV (%)	$\bar{x}$ (cm)	SD (cm)	CV (%)	$\bar{x}$ (cm)	SD (cm)	CV (%)
Waist circumference	WC	69.00	0.27	0.40	71.00	0.0	0.00	68.50	0.55	0.80
Hip circumference	HC	97.50	0.65	0.67	103.00	1.44	1.40	96.00	0.71	0.74
Thigh circumference	TC	54.50	0.35	0.65	57.50	0.57	1.00	57.50	0.89	1.57
Knee circumference	KC	35.50	0.42	1.18	39.00	0.35	0.91	39.50	0.89	2.29

**Table 9:** Differences between mean values of the 3DMSTA and 3DMSIT, 3DMSTA and K3DMSIT and between 3DMSIT and K3DMSIT.

Measurements of 3D Body Models	Symbol	D_3DMSTA-3DMSIT_		D_3DMSTA-K3DMSIT_		D_3DMSIT-K3DMSIT_	
		$\bar{Dx}$ (cm)	$\bar{Dx}$ (%)	$\bar{Dx}$ (cm)	$\bar{Dx}$ (%)	$\bar{Dx}$ (cm)	$\bar{Dx}$ (%)
Waist circumference	WC	−2.20 ^1^	−3.20	0.40	0.58	2.60	3.66
Hip circumference	HC	−5.40	−5.54	1.40	1.46	6.80	6.61
Thigh circumference	TC	−2.80	−5.14	−2.60	−4.55	0.20	0.35
Knee circumference	KC	−3.60	−10.17	−3.70	−9.46	0.10	−0.26

^1^ Note: The minus sign means that the body dimension increased when the body changed posture from a standing to a sitting.

## Data Availability

Not applicable.
